# Frail Patient in Hemodialysis: A New Challenge in Nephrology—Incidence in Our Area, Barcelonès Nord and Maresme

**DOI:** 10.1155/2017/7624139

**Published:** 2017-09-28

**Authors:** Ioana Bancu, Fredzzia Graterol, Jorge Bonal, Pilar Fernández-Crespo, Jessica Garcia, Susana Aguerrevere, Domingo Del Castillo, Josep Bonet

**Affiliations:** ^1^Nephrology Service, Hospital Universitari Germans Trias i Pujol, Badalona, Spain; ^2^Institut d'Investigació en Ciències de la Salut Germans Trias i Pujol (IGTP), Can Ruti Campus, Badalona, Spain; ^3^Agència de Gestió d'Ajuts Universitaris i de Recerca- (AGAUR-) REMAR, Barcelona, Spain; ^4^Red Investigación Renal (REDinREN), Instituto Salud Carlos III, Madrid, Spain; ^5^Diaverum Renal Services, Madrid, Spain

## Abstract

**Introduction:**

Labeling a patient as “frail” may be useful in assessing the prognosis and therapeutic approach.

**Objective:**

The aim of the study is to define a pattern of frailty among our dialysis population, to analyse the incidence and clinical evolution of these patients.

**Materials and Methods:**

We analysed a total of 320 patients with stage V chronic kidney disease (CKD) who were on hemodialysis between September 2014 and September 2015. To define a patient as frail we used the Fried phenotype model, and we added a new criteria-dialysis session length longer than 12 hours/week.

**Results:**

5.6% of the 320 patients were frail. We found statistically significant differences regarding body mass index (BMI), hemoglobin (Hgb), and serum albumin, as well as the ability to perform the basic activities of daily living (*p* < 0.005), ability to ambulate (*p* = 0.01) and perform transfers (*p* < 0.005). We found statistically significant differences between the two groups in terms of hospital admissions (*p* = 0.005) and mortality (*p* < 0.005).

**Conclusion:**

5.6% of the study population were frail, with lower BMI, serum albumin and hemoglobin, lower capacity for basic activities of daily living, ambulation, and transference, as well as higher morbidity and mortality.

## 1. Introduction

The concept of “frail patient” is increasingly used in the clinical practice, without referring to an organic lesion, or to a specific diagnosis, but within the integral vision of the patient.

What has motivated the need to integrate this notion into clinical practice is the aging of the population. Data from the Spanish National Institute of Statistics show that from 1991 to 2001 in Spain the number of people over 85 years has almost doubled, from 449,773 in 1991 to 708,248 in 2001. In the last census in 2011, 1,113,247 people aged over 85 resided in Spain.

Labelling a patient as “frail” may be useful in assessing prognosis, deciding on a therapeutic approach, and planning health resources.

In the field of nephrology the association of chronic kidney disease and senile disease is practically indivisible. The introduction of the renal replacement techniques has undergone changes in its application to the elderly. If elderly patients were not routinely included in the hemodialysis program until the early 1980s [1], there is currently an increase in the age of incident and prevalent patients on hemodialysis. Data from the Registry of Renal Patients of Catalonia [2] confirm that 20% of patients who started hemodialysis in 2014 were older than 80 years. In addition, it is known that in this population the life expectancy after initiating dialysis is of 16 months for patients with an age between 80 and 84 years and 12 months for those of age of 85–89 years.

With a population whose age is clearly increasing, the notion of “frail patient” in hemodialysis is a necessary and essential emerging concept for better clinical care.

There is no consensus on the criteria that should be used to define frailty in patients on dialysis. There are some published papers [3–5] that investigate the frailty in dialysis and they mainly use criteria derived from the Fried frailty phenotype model, without considering characteristics of renal disease and dialysis.

Therefore, the aim of the present study is to define a frailty pattern among our dialysis population, to analyse the incidence and clinical evolution of these patients.

## 2. Materials and Methods

This is a retrospective cross-sectional observational study. Individuals included in the study are patients with stage V chronic kidney disease who were on hemodialysis at 3 Diaverum dialysis centres (Barcelonès Nord and Maresme area) between September 2014 and September 2015. A total of 320 patients were analysed. All the patients approached for the study were enrolled.

Demographic, clinical, and analytical data were collected by reviewing the medical history. We reviewed the hospital admission rate during 1 year.

Data have been collected regarding the walking ability and ability to perform transfers, daily physical activity, and the possibility of performing the basic activities of daily living. The Charlson index and the Karnofsky scale have been calculated.

The Fried phenotype scale was applied to all patients [6].

Frailty was assed using the Fried frailty phenotype scale, the standard definition published by Fried [6] and colleagues in 2001 using data from the Cardiovascular Health Study. Frailty was defined as a clinical syndrome in which three or more of the following criteria were present: unintentional weight loss (10 lbs in past year), self-reported exhaustion, weakness (grip strength), slow walking speed, and low physical activity.

The frail patient on hemodialysis was defined by us with the Fried phenotype scale to which we added as new criteria that they performed more than 12 hours of hemodialysis per week. The current dialysis guidelines [7], DKIQO, recommend 3 dialysis sessions a week of 4 hours with the possibility of increasing dialysis time in special situations such as poor blood pressure control, severe alteration of bone mineral metabolism, and poor tolerance to dialysis, as maintained hypotension during the session that may lead to hyperhydration of patients.

### 2.1. Statistical Analysis

Descriptive statistics were used for all demographic and clinical data. Baseline characteristics were compared between frail and nonfrail patients. Continuous variables and categorical nominal variables with normal distribution were analysed using Student's *t*-test, the Mann–Whitney *U* test, or the Chi-square test, respectively. The normal distribution was assessed using the Kolmogorov-Smirnov test. The level of statistical significance was set at 0.05. All analyses were performed using statistical software for the social sciences (SPSS) version 20.

Logistic regression models were used to assess the association of the mortality with the clinical features and comorbidities.

## 3. Results

### 3.1. Clinical and Laboratory Data of the Entire Population Included in the Study

Clinical data are summarized in Table 1.

Regarding the vascular access, 74.4% of the patients were dialysed through a native arteriovenous fistula, 5.3% through a prosthetic fistula, and the remaining 20.3% through a catheter.

### 3.2. Frail Patient

Using our frailty criteria 5.6% of the patients included in the study were frail.

Using the standard Fried frail phenotype definition 39,06% of our patients met criteria of frailty.

From the total of 320 patients included in the study 20 of them were receiving more than 12 hours of hemodialysis.

We have compared the clinical, analytical, and morbimortality characteristics among frail and nonfrail patients.

Statistically significant differences were found in BMI, hemoglobin (Hgb), and serum albumin between the two groups (Table 2).

As for the associated comorbidities, statistically significant differences were found in the presence of peripheral vasculopathy (*p* = 0.004), cerebrovascular disease (*p* = 0.0079), and depression (*p* = 0.004) between the groups (Table 3).

Statistically significant differences were found in the ability to perform basic activities of daily living (*p* < 0.005) and transfers (*p* < 0.005) and Karnofsky scale (*p* < 0.005) (Table 3).

When analysing vascular access, we detected a higher prevalence of catheter as vascular access in a group of patients defined as frail (Table 4).

We found statistically significant differences between the two groups in terms of hospital admissions (0.77727 admissions/year of frail patients versus 0.2838 admissions per year of nonfrail patients *p* = 0.005). Mortality in the group of frail patients was 20.45%, while in nonfrail patients it was 12.36% (*p* < 0.005).

We analysed the other clinical factors and comorbidities that might be related to hospitalization in our patients.

According to the multivariate analysis in addition to frailty, hospitalization has been associated with diabetes (*p* = 0.035) and peripheral vasculopathy (*p* = 0,018).

## 4. Discussion

In our study, we identified only a 5.6% of the population that fulfilled our frailty criteria: the Fried phenotype definition of frailty widely used in clinical practice, in addition to the increase of more than 12 hours a week of hemodialysis, a criterion also widely used by nephrologists in this type of patients.

The heterogeneity of the concept makes it difficult to estimate its prevalence. Thus, in a review of the recent nephrological literature [8], it is emphasized that the prevalence of frailty among patients affected by CKD can vary from 4 to 59%, depending on the definition applied.

At present, there is no standard definition used for the “frail patient.” In a systematic review of the Cochrane literature [9], the most frequently cited definition is Fried frailty phenotype criteria [6] that defines frailty as a clinical syndrome.

In individuals with CKD the prevalence of frailty increases as renal function declines [10].

In a systematic review aimed to assess frailty prevalence in CKD population, it was highlighted that more than one-third of end stage renal disease patients were frail based on objectively measured Cardiovascular Health Study criteria [11].

We think that the low percentage obtained in our population is due to the strict criteria used (3 or more criteria of the Fried frailty phenotype scale and more than 12 hours a week of hemodialysis). When applying only Fried phenotype scale alone the prevalence of frailty in our population is 39,06%, similar to previously published studies. We believe that it is important to take into account in addition to the phenotypic characteristics criteria related to the dialysis technique when defining a patient as frail. The need for increased dialysis time marks a poor clinical condition and adding this specification when defining a patients as frail may be useful to identify a narrower group of patients requiring increased medical attention.

In our study, age is not related to frailty, indicating that in patients with advanced chronic kidney disease there are other mechanisms involved in the concept of frailty, an aspect that has already been postulated in other studies [12].

The frail patient identified in our study is characterized by a BMI below 25 kg/m2, with a decrease in the ability to perform basic activities of daily living, with a decrease in the ability to ambulate and transfer, and with cerebrovascular disease and associated depression, which has decreased hemoglobin and serum albumin in blood analysis.

Efforts have been made to define the risk factors and mortality associated with dialysis [13]; previous studies have identified diabetes and vascular disease as predictors of mortality [14, 15]. Similarly, our frail patients present vascular disease, and, curiously, cerebrovascular disease and dementia.

Similar results among frail patients in dialysis were published. Frailty among hemodialysis patients is associated with peripheric vasculopathy, cardiac conditions, and low serum albumin [16].

Frail patients in dialysis are more likely to have poor cognitive function at the time of hemodialysis and initiation and worse global cognitive function 1 year after initiation [17].

The association of depression and cerebrovascular disease is known and may be due to a variety of mechanisms including stimulation of the sympathetic nervous system and also involving immune/inflammatory dysfunction. Depressive patients also have poorer health behaviours and depression is more frequent in hypertensive and diabetic patients. Previous papers had shown that patients with depression have an increased cardiovascular risk and an increased risk of morbidity and mortality in the event of a cardiovascular event [18, 19]. The coexistence of depression and frailty is greater than 10%; the presence of depression is being considered a risk factor of frailty [20].

Another remarkable fact in our study is that frail patients significantly use more catheters to perform dialysis sessions. In frail patients, the arteriovenous fistula is often more complicated due to atherosclerosis and/or morbidity that accompanies these patients, justifying this high percentage of catheters in this population. Our results are in agreement with the findings of the literature: catheter use is associated with increased inflammation and therefore mortality in the hemodialysis patient [21, 22].

A common observation is the interaction between the concepts of frailty, dependence, and disability [23, 24]; the emergence of frailty conditions involves greater risk of disability, hospitalization, social isolation, morbidity, and mortality, a hypothesis confirmed in the present study. The frail patients present a high mortality and an increase in the rate of admission and hospital stay.

Interestingly, perceived frailty by the patient or by nephrologist differs from the measured frailty using the Fried frailty phenotype scale and therefore is not accurate to assess frailty [25].

In our study, we found that frail patients compared to nonfrail patients present a higher hospitalization rate and a higher mortality, confirming the importance of having labelled patient as frail for a better clinical supervision.

It was described that in the general population frail older adults, regardless of their comorbidity and disability status, are at twice the risk of mortality and hospitalization [6].

Among dialysis population there is a lack of information regarding the association between frailty, hospitalization, and mortality, although the available results from the literature are indicating that frailty estimated using Fried scale is associated with higher mortality and hospitalization, independent of comorbidity and disability, in adults of all ages [5].

Several papers on frailty's possible causes have been published [26–28]. They can be summarized mainly in factors related to genetics, pathological antecedents, lifestyle, and aging.

In the present study, we performed a registry of the population with chronic kidney disease undergoing hemodialysis in our area. Applying a validated formula for frailty and adding a characteristic of the patient in dialysis—the need to increase dialysis time—we identify those indicators that contribute to or are predictors of frailty.

Although we think that the present work provides useful information on a topic as current in dialysis as frailty, evaluating a new scale of frailty, we admit that our work has limitations: it is an observational study, the frailty was not assessed prior to dialysis initiation, we use a criterion of frailty in which there is no international consensus, and the population sample is only 320 patients.

Therefore, we believe that efforts should be made to reach a common consensus on frailty in hemodialysis and that it is necessary to perform more studies with this consensus criteria and with a larger population sample.

## Figures and Tables

**Table 1:** Clinical characteristics.

Age (years)	70,26 ± 13,85
Gender (M)	59,4%
BMI (kg/m2)	25,93 ± 5,18
Hypertension	94,4%
CVA	85,9%
IHD	71,6%
DM2	39,7%
COPD	10,9%
Liver disease	7,8%
Dementia	4,1%
Neoplastic disease	0,9%

BMI: body mass index; CVA: cerebrovascular accident; IHD: ischemic heart disease; DM2: type 2 diabetes mellitus; COPD: chronic obstructive pulmonary disease; M: male.

**Table 2:** Clinical and analytical characteristics of frail versus nonfrail patients.

Variable	Frail (*n* = 18)	Nonfrail (*n* = 302)	*p*
Age	75,39 ± 11,53	69,85 ± 13,94	0,10
BMI	22,53 ± 3,58	26,16 ± 5,18	0,004
Hgb	10,35 ± 1,31	10,97 ± 1,18	0,032
Albumin	3,61 ± 0,36	3,85 ± 0,29	0,001
Npna	1,12 ± 0,26	10,01 ± 0,26	0,093
CaxP	35,98 ± 10,58	38,34 ± 10,73	0,354
*Ktv*	2,02 ± 0,23	1,88 ± 0,37	0,130

Npna: normalized protein nitrogen appearance; *Ktv*: *K*: urea clearance, *t*: dialysis time, and *V*: distribution volume.

**Table 3:** Associated comorbidities of frail versus nonfrail patients.

Variable	Frail (*n* = 18)	Nonfrail (*n* = 302)	*p*
PV	38,8%	17,21%	0,004
CVD	16,6%	16,55%	0,0079
Depression	38,8%	12,58%	0,004
APBADL	33,33%	76,4%	<0,005
Transfers	38,8%	84,7%	<0,005
Karnofsky	44,4%	95,36%	<0,005

PV: peripheral vasculopathy; CVD: cerebrovascular disease; APBADL: ability to perform basic activities of daily living.

**Table 4:** Vascular access of frail versus nonfrail patients.

Vascular access	Nonfrail	Frail	*p*
Catheter	17,8%	61,6%	<0,005
Native FAVI	77,5%	27,78%	
Prosthetic FAVI	5%	11,11%	

## References

[1] Martín de Francisco A. L. (1998). Hemodiálisis en el anciano. *Nefrología*.

[2] Registre de Malalts Renals de Catalunya: Informe Estadístic 2014

[3] Bao Y., Dalrymple L., Chertow G. M., Kaysen G. A., Johansen K. L. (2012). Frailty, dialysis initiation, and mortality in end-stage renal disease. *Archives of Internal Medicine*.

[4] Johansen K. L., Chertow G. M., Jin C., Kutner N. G. (2007). Significance of frailty among dialysis patients. *Journal of the American Society of Nephrology*.

[5] McAdams-Demarco M. A., Law A., Salter M. L. (2013). Frailty as a novel predictor of mortality and hospitalization in individuals of all ages undergoing hemodialysis. *Journal of the American Geriatrics Society*.

[6] Fried L. P., Tangen C. M., Walston J. (2001). Frailty in older adults: Evidence for a phenotype. *Journals of Gerontology A: Biological Sciences and Medical Sciences*.

[7] KDOQI Clinical Practice Guideline for Hemodialysis Adequacy: 2015 Update 10.1053/j.ajkd.2015.07.015 26498416

[8] Clegg A., Young J., Iliffe S., Rikkert M. O., Rockwood K. (2013). Frailty in elderly people. *The Lancet*.

[9] Carlos Gil A. M., Martínez Pecino F., Molina Linde J. M. (2009). Desarrollo de criterios, indicadores de complejidad y estrategias de manejo en fragilidad Sevilla. *Agencia de Evaluación de Tecnologías Sanitarias de Andalucía*.

[10] Roshanravan B., Khatri M., Robinson-Cohen C. (2012). A prospective study of frailty in nephrology-referred patients with CKD. *American Journal of Kidney Diseases*.

[11] Kojima G. (2017). Prevalence of frailty in end-stage renal disease: a systematic review and meta-analysis. *International Urology and Nephrology*.

[12] Kim J. C., Kalantar-Zadeh K., Kopple J. D. (2013). Frailty and protein-energy wasting in elderly patients with end stage kidney disease. *Journal of the American Society of Nephrology*.

[13] Browne O. T., Allgar V., Bhandari S. (2014). Analysis of factors predicting mortality of new patients commencing renal replacement therapy 10 years of follow-up. *BMC Nephrology*.

[14] Mauri J. M., Clèries M., Vela E., Registry C. R. (2008). Design and validation of a model to predict early mortality in haemodialysis patients. *Nephrology Dialysis Transplantation*.

[15] Pan A., Sun Q., Okereke O. I., Rexrode K. M., Hu F. B. (2011). Depression and risk of stroke morbidity and mortality: A meta-analysis and systematic review. *The Journal of the American Medical Association*.

[16] Kutner N. G., Zhang R., Huang Y., McClellan W. M., Soltow Q. A., Lea J. (2014). Risk factors for frailty in a large prevalent cohort of hemodialysis patients. *American Journal of the Medical Sciences*.

[17] McAdams-Demarco M. A., Tan J., Salter M. L. (2015). Frailty and cognitive function in incident hemodialysis patients. *Clinical Journal of the American Society of Nephrology*.

[18] Goldstein B. I., Carnethon M. R., Matthews K. A. (2015). Major Depressive Disorder and Bipolar Disorder Predispose Youth to Accelerated Atherosclerosis and Early Cardiovascular Disease: A Scientific Statement from the American Heart Association. *Circulation*.

[19] Vaughan L., Corbin A. L., Goveas J. S. (2015). Depression and frailty in later life: A systematic review. *Clinical Interventions in Aging*.

[20] Banerjee T., Kim S. J., Astor B., Shafi T., Coresh J., Powe N. R. (2014). Vascular access type, inflammatory markers, and mortality in incident hemodialysis patients: The choices for healthy outcomes in caring for end-stage renal disease (CHOICE) study. *American Journal of Kidney Diseases*.

[21] Lok C., Floey R. (2013). Vascular access morbidity and mortality. *Clinical Journal of the American Society of Nephrology*.

[22] Brown I., Renwick R., Raphael D. (1995). Frailty: Constructing a common meaning, definition, and conceptual framework. *International Journal of Rehabilitation Research*.

[23] Theou O., Rockwood M. R. H., Mitnitski A., Rockwood K. (2012). Disability and co-morbidity in relation to frailty: How much do they overlap?. *Archives of Gerontology and Geriatrics*.

[24] Drost D., Kalf A., Vogtlander N., van Munster B. C. (2016). High prevalence of frailty in end-stage renal disease. *International Urology and Nephrology*.

[25] Salter M. L., Gupta N., Massie A. B. (2015). Perceived frailty and measured frailty among adults undergoing hemodialysis: A cross-sectional analysis. *BMC Geriatrics*.

[26] Gesualdo G. D., Zazzetta M. S., Say K. G., Orlandi F. D. S. (2016). Factors associated with the frailty of elderly people with chronic kidney disease on hemodialysis. *Ciencia e Saude Coletiva*.

[27] Weibel E. R., Taylor C. R., Hoppeler H. (1991). The concept of symmorphosis: A testable hypothesis of structure-function relationship. *Proceedings of the National Academy of Sciences of the United States of America*.

[28] Botz W. (1984). The tissue syndrome. *Western Journal of Medicine*.

